# *Erratum*: Vol. 65, No. 52

**DOI:** 10.15585/mmwr.mm6603a10

**Published:** 2017-01-27

In the report “Human Rabies — Puerto Rico, 2015,” on page 1476, the first sentence under the Summary heading “What is added by this report?” should have read “A man aged 54 years who was bitten by a mongoose in October **2015** was the first person to acquire rabies from a mongoose in the United States or U.S. territories, confirming mongoose rabies as a public health threat.”

